# Biogenic Amine Content Analysis of Three Chicken-Based Dry Pet Food Formulations

**DOI:** 10.3390/ani13121945

**Published:** 2023-06-10

**Authors:** Nicolò Montegiove, Leonardo Leonardi, Alessio Cesaretti, Roberto Maria Pellegrino, Alessia Pellegrino, Carla Emiliani, Eleonora Calzoni

**Affiliations:** 1 Department of Chemistry, Biology and Biotechnology, Biochemistry and Molecular Biology Section, University of Perugia, Via del Giochetto, 06126 Perugia, Italy; 2 Department of Veterinary Medicine, University of Perugia, Via San Costanzo 4, 06126 Perugia, Italy; 3 Centro di Eccellenza sui Materiali Innovativi Nanostrutturati (CEMIN), University of Perugia, Via del Giochetto, 06126 Perugia, Italy; 4 Independent Researcher, Via Indipendenza 31/A, 06081 Assisi, Italy

**Keywords:** amino acids, nitrogenous compounds, fresh meats, meat meals, kibbles, pet food industry, food handling, microorganisms, bacteria, toxic compounds

## Abstract

**Simple Summary:**

Dry pet food is an often-favored choice by pet owners. In this context, the market offers different options of formulations, mainly meat-meal-based foods, but also fresh-meat-based formulations are more and more often offered as well. In this study, the concentration of biogenic amines and free amino acids in three different chicken-based dry pet food formulations have been analyzed: one of these based on fresh meat, one on meat meal, and one consisting of a mix of these two. The results showed that the fresh-meat-based formulation has a lower quantity of potentially harmful biogenic amines compared to the other formulations, making it the preferable choice when it comes to dry pet food.

**Abstract:**

The pet food market is constantly expanding, and more and more attention is paid to the feeding of pets. Dry foods stand out and are often preferred due to their long shelf life, ease of administration, and low cost. In this context, dry foods are formulated from fresh meats, meat meals, or a mix of the two. These raw materials are often meat not fit for human consumption; they might be subject to contamination and proliferation of microorganisms which, by degrading the organic component, can lead to the formation of undesirable by-products such as biogenic amines. These nitrogenous compounds obtained by decarboxylation of amino acids can therefore be found in high-protein foods, and their ingestion in large quantities can cause intoxication and be harmful. This study aims at analyzing the possible presence of biogenic amines in three different formulations of chicken-based kibbles for pets: one obtained from fresh meat, one from meat meal, and one from a mix of the two. This study is also focused on the presence of free amino acids as they represent the key substrate for decarboxylating enzymes. Mass spectrometry (Q-TOF LC/MS) was used to analyze the presence of biogenic amines and free amino acids. The results show that fresh-meat-based products have a lower content of biogenic amines, and at the same time a higher quantity of free amino acids; on the contrary, meat-meal- and mix-based products have a greater quantity of biogenic amines and a lower concentration of free amino acids, suggesting that there has been a higher microbial proliferation as proved by the total aerobic mesophilic bacteria counts. It is therefore clear that fresh-meat-based kibbles are to be preferred when they are used for preparing dry pet food due to the lowest concentration of biogenic amines.

## 1. Introduction

**Figure 1:**
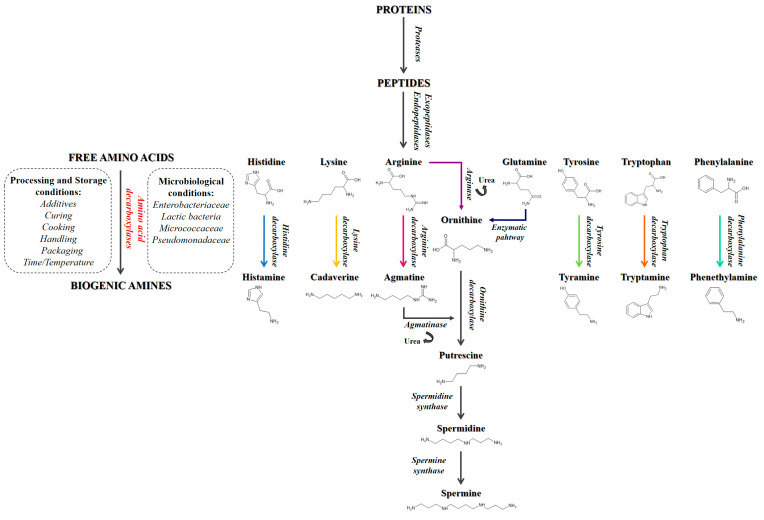
Degradation pathways of amino acids leading to the formation of biogenic amines.

Pet food is a continuously growing sector, with constantly increasing revenues, as more attention is paid to pets, their health, and their nutrition. The market offers a huge choice of pet foods but the best-selling product is dry food with a moisture content of less than 11% [1,2,3,4]. This type of food is often preferred by pet owners as, by virtue of its characteristics, it has a longer storage time than wet foods and can simply be stored at room temperature, allowing consumers to make large provisions and be able to independently feed their pets for a very long period of time; furthermore, it can be formulated to satisfy every kind of different pet nutritional requirements [5,6,7]. Other important factors in choosing this type of diet lie in the ease of administration and low cost, which are often favored by many pet owners. These dry foods represent a valid help in feeding pets, as they are complete foods formulated to provide all the nutrients necessary for the various stages and different needs of the animals’ life, from growth to maintenance. However, due to the inappropriate storage conditions, unwanted compounds, such as biogenic amines, can form, being harmful to animals’ health if taken in large quantities [8,9,10,11,12,13]. Biogenic amines (BAs) are nitrogenous compounds derived from microbial decarboxylation of amino acids (AAs) and from the amination or transamination of ketones and aldehydes following specific microbial enzymatic pathways [8,14,15,16,17,18,19,20,21] (Figure 1).

**Figure 2:**
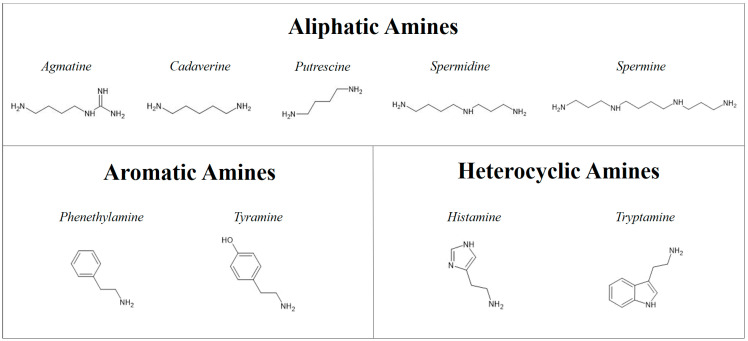
Classification of biogenic amines by their chemical structure.

They can be divided into three groups according to their chemical structure: aliphatic amines (agmatine, cadaverine, putrescine, spermidine, and spermine), aromatic amines (phenethylamine and tyramine), and heterocyclic amines (histamine and tryptamine) [22,23] (Figure 2).

According to the number of amine groups they can be also divided into: monoamines (phenethylamine and tyramine) and polyamines (di-, tri-, tetra-amines) (agmatine, histamine, cadaverine, putrescine, spermidine, spermine, and tryptamine) [24]. Both animals and humans naturally produce BAs; they are involved in natural biological processes such as controlling blood pressure, synaptic transmission, allergic response, and cellular growth [22]. Some BAs may also have beneficial health effects. For example, at physiological concentration, tryptamine and phenethylamine play a role as endogenous neuromodulators of central monoaminergic neurotransmission and also act indirectly as sympathomimetic amines by causing noradrenalin release from sympathetic neurons [25,26]. Additionally, agmatine has been shown to have different functions in the body as it displayed anti-atherosclerosis, anti-inflammatory, antioxidative, neuroprotective, and cell proliferation inhibition properties [27,28,29,30,31]. Several studies have reported its potential therapeutic effect and positive role in vascular, neuronal, and metabolic functions. However, it has also been reported that agmatine can react with nitrites to form nitrosamines, which are carcinogenic compounds. Due to the low activity of arginine decarboxylase in mammals, the amounts of agmatine found in their tissues can only be minimally attributed to *de novo* synthesis by arginine decarboxylase, while high concentrations can result from the diet [32]. The endogenous production of BA can also derive from an excess of undigested amino acids which can be used as a substrate for microbial degradation in the intestine, where different toxic compounds are formed from nitrogen, such as ammonia and amines. In addition to the endogenous concentration of biogenic amines, dietary supplementation of these compounds can cause significant animal health problems [33]. In fact, these nitrogenous compounds can also be found in food as a result of microbial contamination and proliferation, and if BA levels in food or drink reach a critical threshold, they could be harmful to health [34]. The raw materials most commonly used for the production of dry pet food are fresh meats and meat meals deriving from meat industry products not intended for human consumption [35,36,37]. Meat meals are often used due to their high-protein content and are obtained by the rendering process [37,38]. The lack of respect for good pet food manufacturing and handling practices [39,40] could result in a favorable condition for the growth of microorganisms capable of decarboxylation processes [41,42,43]. BAs can therefore be an alarm bell for possible microbial contaminations as they can occur in the final product as a result of microbial decarboxylation processes; this has been observed in dry dog chews and feeds, in sealed packages and in bulk [41,42,43].

The aim of this work is therefore to evaluate the presence of BAs and free AAs (FAAs) in three different formulations of chicken-based dry pet foods: kibbles based on chicken fresh meat (CFM), chicken meat meal (CMM), and a mix of the two (CMix). The analysis of the samples was carried out by quadrupole time-of-flight liquid chromatography/mass spectrometry (Q-TOF LC/MS) in order to quantify the presence of BAs and FAAs in dry pet food. At the same time, a total aerobic mesophilic bacteria count (TAMBC) was performed to evaluate possible microbial contaminations.

These analyses can provide a better understanding of the BA content in the final dry products that could reflect the possible microbial contamination that could occur in the final steps of production and during the conservation [41,42,43]. Moreover, this study can shed light on which is the best choice of chicken-based dry pet food products, from the BA content point of view, to be fed to the pets.

## 2. Materials and Methods

### 2.1. Dry Pet Foods

The dry food for companion animals used in this study was produced by an Italian pet food manufacturer and consists of 3 different formulations based on chicken fresh meat, chicken meat meal, or a mix of the two as the main ingredient, stored at room temperature in sales packages: 12 batches of a kibble formulation made from chicken fresh meat (CFM); 12 batches of a kibble formulation made from a mix of chicken fresh meat and chicken meat meal 1:1 (*w*/*w*) (CMix); 12 batches of a kibble formulation made from chicken meat meal (CMM). Each sample is derived from a different lot of production, produced by the same pet food plant by using uniform production parameters but at slightly different times. Chicken carcasses without legs, wings, and necks were used to prepare chicken meals. Based on the quantity of chicken meat and/or chicken meal, each formulation also comprises varying percentages of the following ingredients: pre-cooked rice, dry rice, beetroot, sorghum, hydrolyzed pork liver, chicken fat, and hydrolyzed chicken proteins. The protein content of the different formulations was evaluated in a previous study [4] and ranges from 23 to 26%. The same extrusion technique was used with all of the formulations, and processing was carried out under the same conditions.

### 2.2. Sample Preparation

A 1.5 mL Eppendorf tube holding 100 mg of dry sample properly weighed was added with 1 mL of methanol containing 2.5 µg/mL of phenylglycine as an internal standard. Tubes were shaken in a Thermomixer T-Shaker (EuroClone, Pero, Italy) for 20 min at 1500 rpm. After the samples were centrifuged at 3300× *g* for 10 min using an Eppendorf 5418 centrifuge (Eppendorf, Hamburg, Germany), the supernatant was placed into vials. An Agilent 6530 Q-TOF LC/MS instrument (Agilent Technologies, Inc., Santa Clara, CA, USA) was then loaded with 0.5 µL of each sample.

### 2.3. Determination of Biogenic Amines and Free Amino Acids

A 150 × 2.1 mm, 3 µm ACME^TM^ Amide C18 column (Phase Analytical Technology LLC, State College, PA, USA) thermostated at 50 °C was utilized using ion-pairing chromatography (IPC) to produce a broad separation of polar metabolite classes. By resorting to a flow of 0.35 mL/min of a binary gradient of 0.3% heptafluorobutyric acid in water (solvent A) and 0.1% formic acid in methanol (solvent B), the separation of biogenic amines (BAs) and free amino acids (FAAs) was accomplished. At first, the employed conditions involved 2% of B for 2 min, followed by a gradient from 2 to 80% of B in 5 min, and finally an isocratic step was carried out for 8 min. Positive ions were detected using the spectrometer in high-resolution full-scan mode. Quantitative data were acquired by external calibration employing a pre-prepared mixture of AAs in pure methanol at concentrations ranging between 0.05 and 2.5 µg/mL. For the study of BA and FAA profiles, samples were then put into an Agilent 6530 Q-TOF LC/MS device (Agilent Technologies, Inc., Santa Clara, CA, USA). Each sample was analyzed in triplicate. The amount of BAs or FAAs in g or mg per kilogram of dry material was used to express the data.

### 2.4. Microbiological Analysis

The determination of the total aerobic mesophilic bacteria count (TAMBC) of the three different dry pet food formulations was performed according to the standard protocol for microbiological analysis of food and animal feeding stuff [44]. Each sample was analyzed in triplicate. Data were expressed as colony-forming units (CFU) per g of dry sample.

### 2.5. Statistical Analysis

All data regarding the analysis of the BA and FAA content and TAMBC of the three different chicken-based dry pet food formulations shown in this study are reported as mean values of the 12 analyzed samples ± standard error of the mean (SEM). To investigate the significance of the differences in the mean values of BA and FAA content and TAMBC of each type of formulation, the one-way ANOVA test was employed. The level of significance for the data was set at *p* < 0.05. All statistical tests were conducted using GraphPad Prism 9.0.0 for Windows (GraphPad Software, San Diego, CA, USA).

## 3. Results

### 3.1. Biogenic Amines

**Figure 3:**
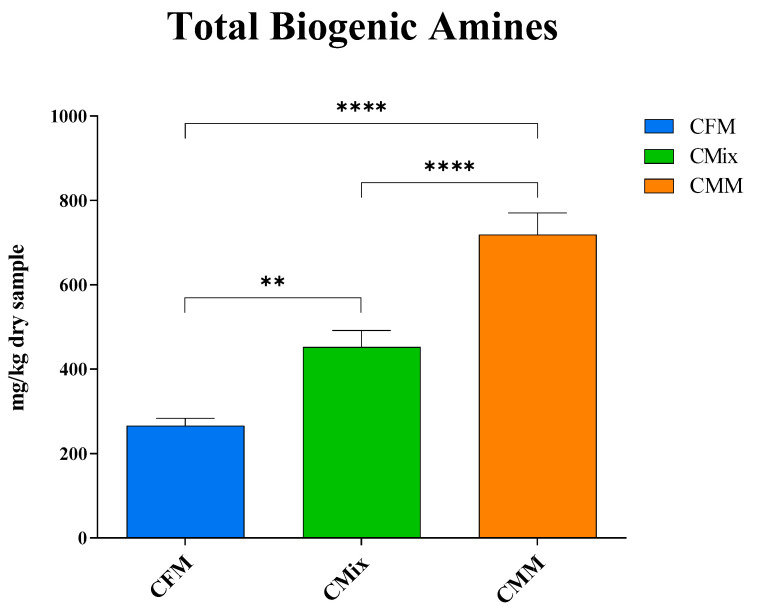
Total biogenic amine content of CFM, CMix, and CMM formulations for companion animal food. Data are reported as mean ± SEM, *n* = 12. ** *p* < 0.01, **** *p* < 0.0001.

The results shown in Figure 3 revealed how the CMM formulation has the highest total BA content (719 mg of BAs/kg of dry sample), followed by the CMix formulation (452 mg of BAs/kg of dry sample) and the CFM formulation (266 mg of BAs/kg of dry sample). The latter is thus the one showing the lowest amount of BAs, resulting in there being less than half of the concentration found in the CMM formulation.

**Figure 4:**
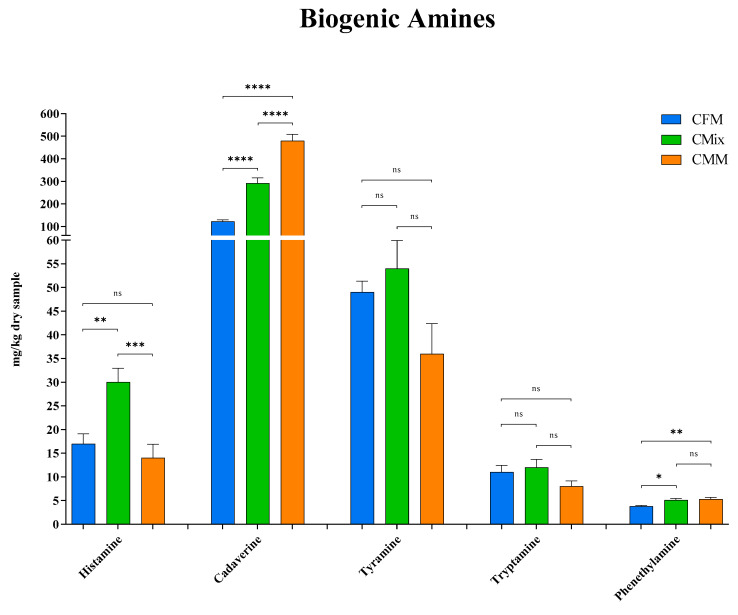
Histamine, cadaverine, tyramine, tryptamine, and phenethylamine content of CFM, CMix, and CMM formulations for companion animal food. Data are reported as mean ± SEM, *n* = 12. ns = difference is not statistically significant, * *p* < 0.05, ** *p* < 0.01, *** *p* < 0.001, **** *p* < 0.0001.

Subsequently, the quantity of the individual BAs detected following the analysis by mass spectrometry was analyzed. The results shown in Figure 4 displayed how the CMix formulation has the highest quantity of histamine (30 mg/kg of dry sample) compared to the CFM (17 mg/kg of dry sample) and CMM (14 mg/kg of dry sample) formulations, which showed an almost halved content of histamine. No significant differences were found between the CFM formulation and the CMM formulation.

As for cadaverine, the CFM formulation showed a significantly lower concentration of this BA (122 mg/kg of dry sample), less than half relative to the CMix formulation (292 mg/kg of dry sample) and about four times less than the CMM formulation (480 mg/kg of dry sample) that instead featured the highest concentration (Figure 4). In addition, for all formulations, cadaverine was found to be the most concentrated BA.

No significant differences were found for tyramine, the mean concentrations of the formulations vary from about 40 to 50 mg per kg of dry sample (49 mg/kg of dry sample for CFM formulation, 54 mg/kg of dry sample for CMix formulation, and 36 mg/kg of dry sample for CMM formulation). Tyramine was also found to be one of the most abundant BA (Figure 4).

The evaluation of tryptamine did not reveal any significant differences as well; the concentrations proved to all be around 10 mg per kg of dry sample (11 mg/kg of dry sample for CFM formulation, 12 mg/kg of dry sample for CMix formulation, and 8 mg/kg of dry sample for CMM formulation) (Figure 4).

In the case of phenethylamine, the CFM formulation displays instead the lowest concentration (3.8 mg/kg of dry sample) compared to the other formulations (5.1 mg/kg of dry sample for CMix formulation and 5.3 mg/kg of dry sample for CMM formulation), while no significant differences were found between the CMix and CMM formulations.

**Figure 5:**
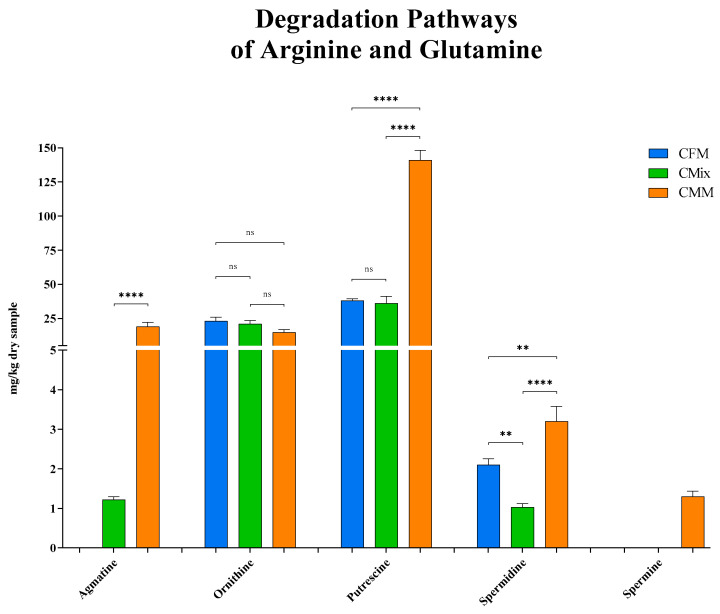
Agmatine, ornithine, putrescine, spermidine, and spermine content of CFM, CMix, and CMM formulations for companion animal food. Data are reported as mean ± SEM, *n* = 12. ns = difference is not statistically significant, ** *p* < 0.01, **** *p* < 0.0001.

As for the degradation pathways of arginine and glutamine, the results shown in Figure 5 highlight that agmatine was not detected in the fresh-meat-based formulation, while it was instead found in the mix formulation (1.22 mg/kg of dry sample) and in the meat-meal-based formulation (19 mg/kg of dry sample) where the highest concentration was measured.

As for the intermediate non-proteinogenic AA ornithine, produced from both arginine and glutamine, the differences found between the three formulations are not statistically significant, with the mean concentrations varying from about 15 to 25 mg per kg of dry sample (23 mg/kg of dry sample for CFM formulation, 21 mg/kg of dry sample for CMix formulation, and 15 mg/kg of dry sample for CMM formulation) (Figure 5).

As far as the degradation pathways of the AAs arginine and glutamine are concerned, putrescine is the one exhibiting the highest concentrations. The CFM and CMix formulations have a putrescine concentration of less than 40 mg per kg of dry sample (38 mg/kg of dry sample for CFM formulation, and 36 mg/kg of dry sample for CMix formulation), and no significant differences were found between them, while the CMM formulation showed the highest concentration, about four times greater compared to the others (141 mg/kg of dry sample) (Figure 5).

As for spermidine, similar trends were observed, with the CMM formulation showing the highest concentration (3.2 mg/kg of dry sample), followed by the CFM formulation (2.1 mg/kg of dry sample) and the CMix formulation (1.03 mg/kg of dry sample) (Figure 5).

Finally, the concentration of spermine was evaluated, but it was only detected in the CMM formulation (1.3 mg/kg of dry sample).

### 3.2. Free Amino Acids

**Figure 6:**
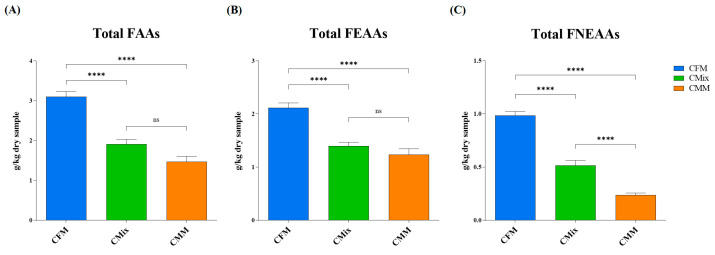
(**A**) Total free amino acid (FAA) content of CFM, CMix, and CMM formulations for companion animal food. (**B**) Total free essential amino acid (FEAA) content of CFM, CMix, and CMM formulations for companion animal food. (**C**) Total free non-essential amino acid (FNEAA) content of CFM, CMix, and CMM formulations for companion animal food. Data are reported as mean ± SEM, *n* = 12. ns = difference is not statistically significant, **** *p* < 0.0001.

The free amino acid (FAA) content of the three different chicken-based dry pet food formulations was also evaluated. The results shown in Figure 6A revealed how the CFM formulation has the highest total (FAA) content (3.10 g of FAAs/kg of dry sample), followed by the CMix formulation (1.91 g of FAAs/kg of dry sample) and the CMM formulation (1.47 g of FAAs/kg of dry sample). The latter is the one that exhibited the least amount of FAAs, showing less than half of the quantity found in the CFM formulation.

At the same time, the content of the total free essential amino acids (FEAAs) and the total free non-essential amino acids (FNEAAs) was evaluated. The results in Figure 6B,C showed that the CFM formulation has the highest content of total FEAAs (2.11 g of total FEAAs/kg of dry sample) compared to the other formulations, which have almost half the quantity (1.39 g of total FEAAs/kg of dry sample for the CMix formulation and 1.23 g of total FEAAs/kg of dry sample for the CMM formulation). No significant differences were found between the CMix and the CMM formulations (Figure 6B). As far as the total FNEAAs content is concerned, the CFM formulation again showed the highest concentration (0.98 g of total FNEAAs/kg of dry sample), followed by the CMix formulation (0.52 g of total FNEAAs/kg of dry sample) and the CMM formulation (0.24 g of total FNEAAs/kg of dry sample), which has a quantity more than four times lower than CFM (Figure 6C).

**Figure 7:**
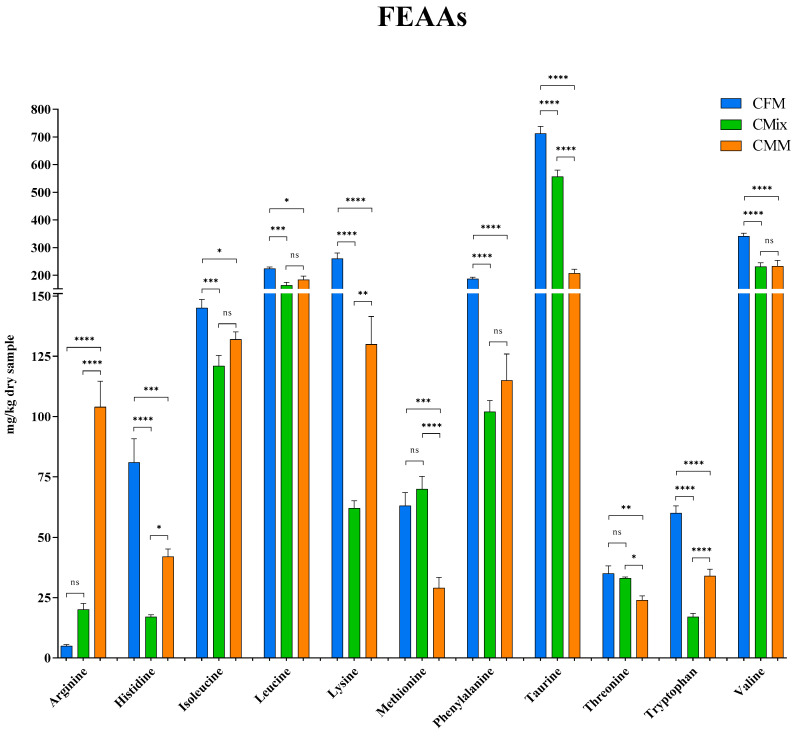
Free essential amino acid (FEAA) content of CFM, CMix, and CMM formulations for companion animal food. Data are reported as mean ± SEM, *n* = 12. ns = difference is not statistically significant, * *p* < 0.05, ** *p* < 0.01, *** *p* < 0.001, **** *p* < 0.0001.

**Figure 8:**
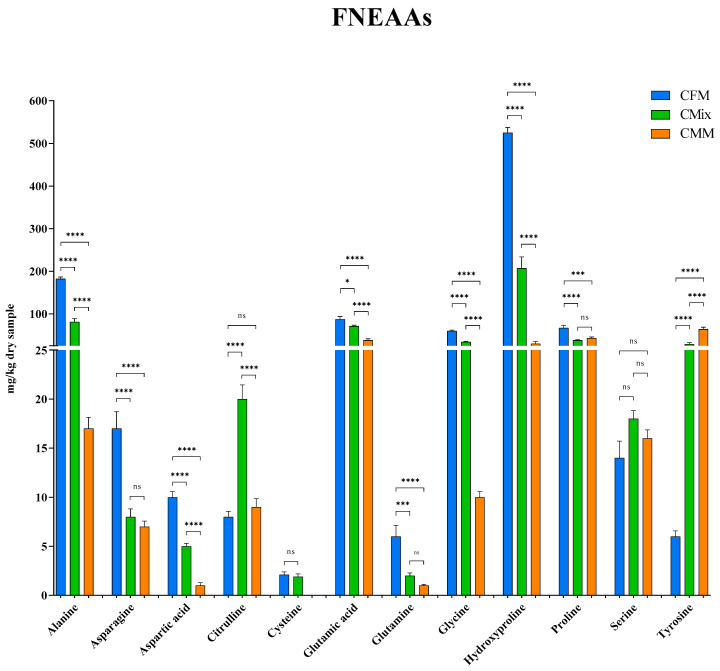
Free non-essential amino acid (FNEAA) content of CFM, CMix, and CMM formulations for companion animal food. Data are reported as mean ± SEM, *n* = 12. ns = difference is not statistically significant, * *p* < 0.05, *** *p* < 0.001, **** *p* < 0.0001.

Here, the individual FAAs found in the three different formulations are also reported: Figure 7 shows the FEAAs, while Figure 8 shows the FNEAAs.

With the exception of arginine, methionine, and threonine, all other FEAAs are more concentrated in the CFM formulation compared to the other formulations (Figure 7). Noteworthy is, for example, lysine with a concentration of 260 mg/kg of dry sample in the CFM formulations as opposed to 130 mg/kg of dry sample in the CMM formulation, which thus features half the concentration, and 62 mg/kg of dry sample in the CMix formulation, being more than four times lower. As far as the branched-chain AAs (BCAAs) are concerned, the CFM formulation again shows the highest concentrations compared to the other formulations (145 mg of isoleucine/kg of dry sample, 224 mg of leucine/kg of dry sample, and 341 mg of valine/kg of dry sample). The results concerning phenylalanine, one of the most abundant FEAA, again revealed a higher concentration in the CFM formulation with 187 mg/kg of dry sample, followed by CMM and CMix formulations, where instead no significant differences are found, with 115 mg/kg of dry sample and 102 mg/kg of dry sample, respectively. As for taurine, a sulfur-containing AA, it is the most present FEAA for all formulations with 712 mg/kg of dry sample for the CFM formulation, 557 mg/kg of dry sample for the CMix formulation, and 207 mg/kg of dry sample for the CMM formulation, where the quantity is more than three times less compared to the fresh-meat-based formulation.

The content of individual FNEAAs was subsequently investigated, Figure 8 shows the AAs detected. As is the case with FEAAs, among the FNEAAs the most common trend also shows the CFM formulation having the highest concentrations (Figure 8). Of particular emphasis is hydroxyproline which is the most represented FNEAAs in the CFM formulation (525 mg/kg of dry sample), being more than double that in the CMix formulation (207 mg/kg of dry sample) and about eighteen times more concentrated than in the CMM formulation (30 mg/kg of dry sample). Alanine, glutamic acid, glycine, and proline also respect the same trend with the following concentrations detected for the CFM formulation: 182 mg/kg of dry sample, 87 mg/kg of dry sample, 60 mg/kg of dry sample, and 67 mg/kg of dry sample, respectively; in the peculiar case of tyrosine, it is the CMM formulation to show instead the highest concentration, with a content of 64 mg/kg of dry sample, so that tyrosine also becomes the most abundant FNEAA in this formulation.

### 3.3. Microbiological Analysis

The total aerobic mesophilic bacteria count (TAMBC) of dry final products showed that the CMM formulation had the highest number of colonies (2900 CFU/g of dry sample), followed by the CMix formulation (1500 CFU/g of dry sample) and the CFM formulation (900 CFU/g of dry sample) (Table 1). The CFUs detected in the CMM formulation are about two times higher compared to the CMix formulation (*p* < 0.0001) and about three times higher than in the CFM formulation (*p* < 0.0001), while the CFUs found in the CMix formulation are almost double compared to the CFM formulation (*p* < 0.001).

## 4. Discussion

Much attention is paid to the health of pets and their nutrition, but there is not always full awareness of what is offered to animals and of the possible problems related to the presence of toxic substances. This study is thus focused on the presence of biogenic amines (BAs), microbial decarboxylation products that can be harmful to animal health. The analysis of the three different dry chicken-based formulations showed that these substances can be present in the finished products at different concentrations, with the chicken meat meal (CMM) formulation featuring the greatest quantity (Figure 3). This could be related to the possible microbial contamination and proliferation occurring during the handling and storage of the final product [41,42,43]. More specifically, as far as histamine is concerned, a BA that derives from the decarboxylation of the essential amino acid (EAA) histidine (Figure 1) [9], this nitrogenous compound was detected in all three different formulations (Figure 4), and its intake is mainly involved with the development of allergic phenomena and toxic reactions [45,46,47,48,49,50,51]. Currently, there are no guidelines regarding the concentration of BAs in foods; however, as far as histamine is concerned, literature studies have reported that quantities higher than 100 mg/kg can generally be considered potentially toxic, while other studies report harmful effects already starting from 50 mg/kg, recommending that the daily intake should not exceed this limit [52,53,54,55,56]. In any case, none of the formulations analyzed exceed these values. As for cadaverine, a BA deriving from the decarboxylation of the EAA lysine (Figure 1) [45,52,57], the CMM formulation shows the highest concentration (Figure 4), which also represents the highest value among all BAs studied; cadaverine could be a risk to the animal’s health, as also this amine was found be toxic [13,58,59]. The presence of this BA could be due to the possible contaminations that could occur during the production or storage processes of the final product, as highlighted by the highest number of CFUs found in the CMM formulation (Table 1). In fact, it has been observed in the literature that there may be bacterial contamination during storage [41,42,43], which could plausibly lead to the formation of BAs. Conversely, the concentration of the parent EAA lysine was found to be the highest in the formulation consisting of chicken fresh meat (Figure 7), a result that could be reasonably in line with its lower decarboxylation to cadaverine. However, all the CFU values found for all the formulations remained below the limit values of TAMBC deemed acceptable for pet food [42,43,60], taking into account that there are currently no legal provisions that specify the permissible level of total mesophilic bacteria in these types of food. As far as tyramine and tryptamine are concerned, decarboxylation products of the non-EAA (NEAA) tyrosine and EAA tryptophan, respectively (Figure 1) [45,52,57], the concentrations found were similar between the different formulations (Figure 4); this could be justified by the same degree of bacterial decarboxylation of these two AAs. These BAs could be harmful to pet health as tyramine can be toxic for the intestinal cells, being reported that even concentrations lower than 100 mg/kg can cause adverse effects [54,58,59], while tryptamine can display neurotoxic effects such as hallucinations and serotonergic neurotoxicity [61,62]. As far as phenethylamine is concerned, a decarboxylation product of the EAA phenylalanine (Figure 1) [45,52,57], what has been observed (Figure 4) could be justified by the fact that there are more decarboxylation processes affecting phenylalanine in the formulations containing meat meal or the mix, where the concentration of free phenylalanine was also found to be lower (Figure 7). Even if found in low concentrations, this amine could still have toxic effects, as it or its derivatives can accumulate in kidneys, triggering nephrotoxic phenomena [63]; in fact, it is reported that even concentrations lower than 30 mg/kg show toxicity effects [54,55].

As far as the microbial degradation pathways of arginine and glutamine are concerned, these are two routes starting from different AAs that finally converge into a common path (Figure 1). The intermediate non-proteinogenic AA ornithine is the point of contact between the two pathways [11,52,57,64]; in fact, following distinct enzymatic processes, it can be produced starting from both AAs, finally leading to the formation of putrescine, a BA involved in different toxic complications [13,58,65,66]. The latter BA can be transformed into two secondary BAs, spermidine and spermine, that may be neurotoxic and can cause several health problems such as emaciation, convulsions, aggressiveness phenomena, and paralysis symptoms [58]. Further to this, arginine can also be initially decarboxylated to agmatine [32,45,67], a BA that does not directly cause toxicity phenomena but can be further degraded to putrescine, reconnecting with the pathway described above [13,58,65,66].

The three different formulations showed a completely different trend as regards the metabolites involved in the degradation pathways of arginine and glutamine. Basically, it has been found that meat-meal-based formulation always has higher amounts of BAs (Figure 5). Conversely, it came to light that the fresh-meat-based formulation is the one that shows the least concentrations, and the fact that agmatine and spermine have not been detected in the CFM formulation suggests that there was a lower degree of bacterial proliferation, as also highlighted by the total aerobic mesophilic bacteria count (TAMBC) (Table 1). It should be noted that the CMM formulation is the only one to reveal spermine, the last BA of the degradation pathways of arginine and glutamine, suggesting that there has been a greater microbial proliferation in this kind of formulation, as disclosed by the TAMBC (Table 1), that could justify a more advanced degradation process that would have led to the formation of this secondary BA.

As far as the content of free AAs (FAAs) is concerned, the fact that they are more abundant in the fresh-meat-based formulation (Figure 6A), where the content of BAs is instead lower (Figure 3), could therefore be explained by lower microbial contamination or proliferation (Table 1). Of particular note is the higher content in the CFM formulation of both free EAAs (FEAAs) and free non-EAAs (FNEAAs) (Figure 6, Figure 7 and Figure 8), which could offer a greater substrate for decarboxylation processes; however, the lower bacterial load found in the CFM formulation (Table 1) could justify fewer BAs to be found in the finished product (Figure 3).

Reducing microbial contamination is therefore imperative to reduce the formation of BAs, and correct material handling and conservation procedures are necessary to counteract microbial proliferation. It has already been seen that sterile meat is devoid of BAs, while their concentration increases proportionally to the increase in microbial flora [68,69]. Although some BAs occur naturally at low concentrations as a result of cellular metabolism, the pool of BAs in the final product is often greater. The results of this study highlight how the CMM formulation is the one having the highest concentration of BAs (Figure 3), probably due to higher microbial contamination or proliferation as highlighted by the TAMBC (Table 1). It is therefore necessary to control all those processes and factors that can promote microbial growth and the formation of BAs to limit the presence of these nitrogenous compounds in the final product. From this, it is clear how a high concentration of BAs in finished products could indicate contaminations likely occurring during the final steps of production or subsequent storage [41,42,43]. Higher BA content may result in a possible poor state of health of the animal, in that BAs are responsible for numerous adverse effects for the organism, from allergic reactions involving histamine [9,10,11,46,47,48,49,50,51] to numerous other types of toxic effects of which cadaverine, tyramine, tryptamine, phenethylamine, putrescine, spermidine, and spermine are responsible for [10,11,12,13,61,62,63].

## 5. Conclusions

This study focused on the evaluation of the concentration of biogenic amines in three different formulations of chicken-based kibbles, one made of chicken fresh meat (CFM), one of chicken meat meal (CMM), and one consisting of a mix of the two (CMix). The results showed that the fresh-meat-based formulations are those showing the lowest amount of biogenic amines, as opposed to what was found in the meat-meal-based formulations which instead showed the highest quantity among the three formulations analyzed. The analysis of free amino acids, both essential and non-essential, showed that the CFM formulation is the one having the largest amount, as opposed to the CMM formulation. This trend could justify the greater content of biogenic amines in the CMM formulation, which probably underwent greater microbial decarboxylation processes due to greater microbial contamination and proliferation as proved by the total aerobic mesophilic bacteria count. This aspect makes the CMM formulation of lower quality due to the biogenic amine content which can potentially pose a risk to animal health. At the same time, it could be deduced that the CFM formulation, maintaining higher concentrations of the pool of free amino acids, has been subject to fewer amino acid decarboxylation processes, as suggested by the minor bacterial contamination. Considering what has been observed, it is highlighted that, as a consequence of the reduced microbial contamination and proliferation likely occurring during the final steps of production and subsequent handling processes, the fresh-meat-based formulation is to be preferred when it comes to the choice of dry pet food by virtue of the lower content of potentially toxic biogenic amines.

## Figures and Tables

**Table 1:** Total aerobic mesophilic bacteria count (TAMBC) of CFM, CMix, and CMM formulations for companion animal food. Data are reported as mean ± SEM, *n* = 12.

Dry Pet Food	TAMBC (CFU/g of Dry Sample)
CFM	900 ± 40
CMix	1500 ± 70
CMM	2900 ± 160

## Data Availability

Data is contained within the article.

## References

[1] Gibson M.W., Sajid A. (2013). Pet Food Processing: Understanding Transformations in Starch during Extrusion and Baking. Cereal Foods World.

[2] Aldrich G. (2006). Rendered Products in Pet Food. Essent. Render..

[3] Zicker S.C. (2008). Evaluating Pet Foods: How Confident Are You When You Recommend a Commercial Pet Food?. Top. Companion Anim. Med..

[4] Montegiove N., Calzoni E., Cesaretti A., Pellegrino R.M., Emiliani C., Pellegrino A., Leonardi L. (2022). The Hard Choice about Dry Pet Food: Comparison of Protein and Lipid Nutritional Qualities and Digestibility of Three Different Chicken-Based Formulations. Animals.

[5] Di Donfrancesco B., Koppel K., Swaney-Stueve M., Chambers E. (2014). Consumer Acceptance of Dry Dog Food Variations. Animals.

[6] Morelli G., Stefanutti D., Ricci R. (2021). A Survey among Dog and Cat Owners on Pet Food Storage and Preservation in the Households. Animals.

[7] Rombach M., Dean D.L. (2021). It Keeps the Good Boy Healthy from Nose to Tail: Understanding Pet Food Attribute Preferences of US Consumers. Animals.

[8] Learey J.J., Crawford-Clark S., Bowen B.J., Barrow C.J., Adcock J.L. (2018). Detection of Biogenic Amines in Pet Food Ingredients by RP-HPLC with Automated Dansyl Chloride Derivatization. J. Sep. Sci..

[9] Kovacova-Hanuskova E., Buday T., Gavliakova S., Plevkova J. (2015). Histamine, Histamine Intoxication and Intolerance. Allergol. Immunopathol..

[10] del Rio B., Redruello B., Linares D.M., Ladero V., Fernandez M., Martin M.C., Ruas-Madiedo P., Alvarez M.A. (2017). The Dietary Biogenic Amines Tyramine and Histamine Show Synergistic Toxicity towards Intestinal Cells in Culture. Food Chem..

[11] Montegiove N., Calzoni E., Cesaretti A., Alabed H., Pellegrino R.M., Emiliani C., Pellegrino A., Leonardi L. (2020). Biogenic Amine Analysis in Fresh Meats and Meat Meals Used as Raw Materials for Dry Pet Food Production. Sci. Bull. Ser. F Biotechnol..

[12] Linares D.M., del Rio B., Redruello B., Ladero V., Martin M.C., Fernandez M., Ruas-Madiedo P., Alvarez M.A. (2016). Comparative Analysis of the in Vitro Cytotoxicity of the Dietary Biogenic Amines Tyramine and Histamine. Food Chem..

[13] Del Rio B., Redruello B., Linares D.M., Ladero V., Ruas-Madiedo P., Fernandez M., Martin M.C., Alvarez M.A. (2019). The Biogenic Amines Putrescine and Cadaverine Show in Vitro Cytotoxicity at Concentrations That Can Be Found in Foods. Sci. Rep..

[14] Suzzi G., Torriani S. (2015). Editorial: Biogenic Amines in Foods. Front. Microbiol..

[15] Tabanelli G. (2020). Biogenic Amines and Food Quality: Emerging Challenges and Public Health Concerns. Foods.

[16] Schirone M., Esposito L., D’Onofrio F., Visciano P., Martuscelli M., Mastrocola D., Paparella A. (2022). Biogenic Amines in Meat and Meat Products: A Review of the Science and Future Perspectives. Foods.

[17] Martuscelli M., Esposito L., Mastrocola D. (2021). Biogenic Amines’ Content in Safe and Quality Food. Foods.

[18] Vasconcelos H., Coelho L.C.C., Matias A., Saraiva C., Jorge P.A.S., de Almeida J.M.M.M. (2021). Biosensors for Biogenic Amines: A Review. Biosensors.

[19] Vasconcelos H.C.A.S.G., Marques Martins de Almeida J.M., Mendes J.P., Dias B., Jorge P.A.d.S., Saraiva C.M.T., Coelho L.C.C. (2022). Optical Biosensor for the Detection of Biogenic Amines. IEEE Sens. J..

[20] Vasconcelos H., de Almeida J.M.M.M., Matias A., Saraiva C., Jorge P.A.S., Coelho L.C.C. (2021). Detection of Biogenic Amines in Several Foods with Different Sample Treatments: An Overview. Trends Food Sci. Technol..

[21] Altafini A., Roncada P., Sonfack G.M., Guerrini A., Romeo G.A., Fedrizzi G., Caprai E. (2022). Occurrence of Histamine in Commercial Cat Foods under Different Storage Conditions. Vet. Sci..

[22] EFSA Panel on Biological Hazards (BIOHAZ) (2011). Scientific Opinion on Risk Based Control of Biogenic Amine Formation in Fermented Foods. EFSA J..

[23] Li B., Lu S. (2020). The Importance of Amine-Degrading Enzymes on the Biogenic Amine Degradation in Fermented Foods: A Review. Process. Biochem..

[24] Erdag D., Merhan O., Yildiz B. (2018). Biochemical and Pharmacological Properties of Biogenic Amines. Biogenic Amines.

[25] del Rio B., Redruello B., Fernandez M., Martin M.C., Ladero V., Alvarez M.A. (2020). The Biogenic Amine Tryptamine, Unlike β-Phenylethylamine, Shows in Vitro Cytotoxicity at Concentrations That Have Been Found in Foods. Food Chem..

[26] Berry M.D. (2004). Mammalian Central Nervous System Trace Amines. Pharmacologic Amphetamines, Physiologic Neuromodulators. J. Neurochem..

[27] Raasch W., Schäfer U., Chun J., Dominiak P. (2001). Biological Significance of Agmatine, an Endogenous Ligand at Imidazoline Binding Sites. Br. J. Pharm..

[28] Sezer A., Güçlü B., Kazancı B., Çakır M., Çoban M.K. (2014). Neuroprotective Effects of Agmatine In Experimental Peripheral Nerve Injury In Rats: A Prospective Randomized and Placebo-Controlled Trial. Sıçanlarda Deneysel Periferik Sinir Yaralanmasında Agmatinin Nöroprotektif Etkileri: Prospektif, Randomize, Plasebo Kontrollü Çalışma.

[29] Liu G., Mei H., Chen M., Qin S., Li K., Zhang W., Chen T. (2019). Protective Effect of Agmatine against Hyperoxia-Induced Acute Lung Injury via Regulating LncRNA Gadd7. Biochem. Biophys. Res. Commun..

[30] Li X., Liu Z., Jin H., Fan X., Yang X., Tang W., Yan J., Liang H. (2014). Agmatine Protects against Zymosan-Induced Acute Lung Injury in Mice by Inhibiting NF-*κ*B-Mediated Inflammatory Response. BioMed Res. Int..

[31] Li Y.F., Gong Z.H., Cao J.B., Wang H.L., Luo Z.P., Li J. (2003). Antidepressant-like Effect of Agmatine and Its Possible Mechanism. Eur. J. Pharmacol..

[32] Galgano F., Caruso M., Condelli N., Favati F. (2012). Focused Review: Agmatine in Fermented Foods. Front. Microbiol..

[33] Fan P., Song P., Li L., Huang C., Chen J., Yang W., Qiao S., Wu G., Zhang G., Ma X. (2017). Roles of Biogenic Amines in Intestinal Signaling. Curr. Protein Pept. Sci..

[34] Ladero V., Calles-Enriquez M., Fernandez M., Alvarez M.A. (2010). Toxicological Effects of Dietary Biogenic Amines. Curr. Nutr. Food Sci..

[35] Montegiove N., Pellegrino R.M., Emiliani C., Pellegrino A., Leonardi L. (2021). An Alternative Approach to Evaluate the Quality of Protein-Based Raw Materials for Dry Pet Food. Animals.

[36] Montegiove N., Calzoni E., Cesaretti A., Alabed H., Pellegrino R.M., Emiliani C., Pellegrino A., Leonardi L. (2020). Comprehensive Evaluation of Lipidic Content in Dry Pet Food Raw Materials: Comparison between Fresh Meats and Meat Meals. Sci. Bull. Ser. F Biotechnol..

[37] Thompson A. (2008). Ingredients: Where Pet Food Starts. Top. Companion Anim. Med..

[38] Montegiove N., Calzoni E., Cesaretti A., Pellegrino R.M., Emiliani C., Pellegrino A., Leonardi L. (2021). Soluble Protein Content Assessment in Dry Pet Food Raw Materials: Comparison between Fresh Meat and Meat Meal Formulations. Sci. Bull. Ser. F Biotechnol..

[39] FEDIAF (2018). Guide to Good Practice for the Manufacture of Safe Pet Foods.

[40] Leiva A., Molina A., Redondo-Solano M., Artavia G., Rojas-Bogantes L., Granados-Chinchilla F. (2019). Pet Food Quality Assurance and Safety and Quality Assurance Survey within the Costa Rican Pet Food Industry. Animals.

[41] Kępińska-Pacelik J., Biel W. (2021). Microbiological Hazards in Dry Dog Chews and Feeds. Animals.

[42] Girio T.M.S., Filho A.N., Junior O.D.R., Amaral L.A., Girio R.J.S. (2012). Microbiological Quality of Dog Feed Sold in Sealed Packages and in Bulk. Ars Vet..

[43] Kazimierska K., Biel W., Witkowicz R., Karakulska J., Stachurska X. (2021). Evaluation of Nutritional Value and Microbiological Safety in Commercial Dog Food. Vet. Res. Commun..

[44] (2007). Microbiology of Food and Animal Feeding Stuffs—General Requirements and Guidance for Microbiological Examinations.

[45] Ruiz-Capillas C., Jiménez-Colmenero F. (2005). Biogenic Amines in Meat and Meat Products. Crit. Rev. Food Sci. Nutr..

[46] White M.V. (1990). The Role of Histamine in Allergic Diseases. J. Allergy Clin. Immunol..

[47] Leonardi A. (2000). Role of Histamine in Allergic Conjunctivitis. Acta Ophthalmol. Scand..

[48] Taylor S.L., Eitenmiller R.R. (1986). Histamine Food Poisoning: Toxicology and Clinical Aspects. CRC Crit. Rev. Toxicol..

[49] Maintz L., Novak N. (2007). Histamine and Histamine Intolerance. Am. J. Clin. Nutr..

[50] Altafini A., Roncada P., Guerrini A., Sonfack G.M., Accurso D., Caprai E. (2022). Development of Histamine in Fresh and Canned Tuna Steaks Stored under Different Experimental Temperature Conditions. Foods.

[51] Visciano P., Schirone M., Paparella A. (2020). An Overview of Histamine and Other Biogenic Amines in Fish and Fish Products. Foods.

[52] ten Brink B., Damink C., Joosten H.M., Huis in ’t Veld J.H. (1990). Occurrence and Formation of Biologically Active Amines in Foods. Int. J. Food Microbiol..

[53] Papavergou E.J., Savvaidis I.N., Ambrosiadis I.A. (2012). Levels of Biogenic Amines in Retail Market Fermented Meat Products. Food Chem..

[54] Feddern V., Mazzuco H., Fonseca F.N., Lima G.J.M.M. (2019). de A Review on Biogenic Amines in Food and Feed: Toxicological Aspects, Impact on Health and Control Measures. Anim. Prod. Sci..

[55] Wójcik W., Łukasiewicz-Mierzejewska M., Damaziak K., Bień D. (2022). Biogenic Amines in Poultry Meat and Poultry Products: Formation, Appearance, and Methods of Reduction. Animals.

[56] Danchuk A.I., Komova N.S., Mobarez S.N., Doronin S.Y., Burmistrova N.A., Markin A.V., Duerkop A. (2020). Optical Sensors for Determination of Biogenic Amines in Food. Anal. Bioanal. Chem..

[57] Geornaras I., Dykes G.A., von Holy A. (1995). Biogenic Amine Formation by Poultry-Associated Spoilage and Pathogenic Bacteria. Lett. Appl. Microbiol..

[58] Til H.P., Falke H.E., Prinsen M.K., Willems M.I. (1997). Acute and Subacute Toxicity of Tyramine, Spermidine, Spermine, Putrescine and Cadaverine in Rats. Food Chem. Toxicol..

[59] Lewis R.A. (1998). Lewis’ Dictionary of Toxicology.

[60] Kukier E., Goldsztejn M., Grenda T., Kwiatek K., Wasyl D., Hoszowski A. (2012). Microbiological Quality of Compound Feed Used in Poland. J. Vet. Res..

[61] Tittarelli R., Mannocchi G., Pantano F., Saverio Romolo F. (2015). Recreational Use, Analysis and Toxicity of Tryptamines. Curr. Neuropharmacol..

[62] Mousseau D.D. (1993). Tryptamine: A Metabolite of Tryptophan Implicated in Various Neuropsychiatric Disorders. Metab. Brain Dis..

[63] Mossoba M.E., Vohra S.N., Wiesenfeld P.L., Sprando R.L. (2016). Nephrotoxicity of Combining 2-Phenethylamine and N, N-Dimethyl-β-Phenethylamine. Appl. Vitr. Toxicol..

[64] Wu G., Haynes T.E., Li H., Meininger C.J. (2000). Glutamine Metabolism in Endothelial Cells: Ornithine Synthesis from Glutamine via Pyrroline-5-Carboxylate Synthase. Comp. Biochem. Physiol. Part A Mol. Integr. Physiol..

[65] de Vera N., Serratosa J., Artigas F., Martínez E. (1992). Toxic Effects of Putrescine in Rat Brain: Polyamines Can Be Involved in the Action of Excitotoxins. Amino Acids.

[66] Wunderlichová L., Buňková L., Koutný M., Jančová P., Buňka F. (2014). Formation, Degradation, and Detoxification of Putrescine by Foodborne Bacteria: A Review. Compr. Rev. Food Sci. Food Saf..

[67] Halaris A., Plietz J. (2007). Agmatine: Metabolic pathway and spectrum of activity in brain. CNS Drugs.

[68] Bardócz S. (1995). Polyamines in Food and Their Consequences for Food Quality and Human Health. Trends Food Sci. Technol..

[69] Slemr J., Beyermann K. (1985). Concentration Profiles of Diamines in Fresh and Aerobically Stored Pork and Beef. J. Agric. Food Chem..

